# Fissure of the Anus

**Published:** 1877-04

**Authors:** 


Fissure of the Anus.—In place of sudden dilatation or complete incision, Prof. Gosselin, (*Union Med.*, Jan. 16, 1877) combines the methods, in part. He introduces the index finger every day into the anus, until he is able to see the upper part of the fissure. He next incises this, but only dividing about a fourth of the sphincter ani. He then introduces daily a tent smeared with cerate of extract of rhatany. This method has proved very successful in his hands.—*Med. Times and Gaz.*, Jan. 27, 1877.

